# Creation and Implementation of a Multidisciplinary Pediatric Hematopoietic Stem Cell Transplant Discharge Coordination Program

**DOI:** 10.3390/nursrep15060202

**Published:** 2025-06-04

**Authors:** Jessica D. Murphy, Kathryn Duke, Cambree J. Fillis, Heather J. Symons

**Affiliations:** 1 School of Nursing, Johns Hopkins University, Baltimore, MD 21205, USA; 2 The Johns Hopkins Hospital Children’s Center, Baltimore, MD 21287, USA; kpurcel6@jhmi.edu (K.D.); cfillis1@jh.edu (C.J.F.); 3 Pediatric Blood and Marrow Transplantation Program, Division of Pediatric Oncology, Department of Oncology, Sidney Kimmel Comprehensive Cancer Center, Johns Hopkins University School of Medicine, Baltimore, MD 21287, USA; hsymons2@jhmi.edu

**Keywords:** nursing, care coordination, hospital discharge, pediatric, oncology, BMT, HSCT, stem cell, transplant

## Abstract

**Background/Objectives:** Hospital discharge of pediatric hematopoietic stem cell transplant (HSCT) patients is complex and requires multidisciplinary efforts to ensure patients/caregivers are prepared for transition to the outpatient setting. This period is tenuous as patients are medically complex, immunocompromised, and required to take several medications requiring dose titration. Miscommunication or decreased preparedness for discharge can place patients at risk for life-threatening complications. An integrative review was performed to evaluate the current literature on discharge coordination best practices for pediatric HSCT, revealing a scarcity of data. Taking into account this minimal literature and the lack of an established process at our center, this article details the development and implementation of a multidisciplinary care coordination program for pediatric HSCT patients following hospital discharge, aiming to establish a standardized approach and thus improve caregiver readiness for discharge. **Methods:** A group of physicians, advanced practice nurses, registered nurses, and pharmacists developed a comprehensive approach to pediatric HSCT discharge coordination. Interventions included standardized education, checklist integrated into the electronic medical record, 24 h rooming-in period, and personalized pharmacist follow-up. Surveys were provided to caregivers to assess discharge readiness and ongoing medication adherence. **Results:** This quality improvement project demonstrated feasibility via successful implementation for 12 patients. Compared to a nine-patient pre-implementation group, there was no statistically significant difference in perceived readiness. Medication adherence was unable to be evaluated. Clinical significance was anecdotally appreciated by the medical care team, with improved organization, collaboration, and communication. **Conclusions:** A new pediatric HSCT discharge coordination program was created and successfully implemented. More literature on best practices is needed.

## 1. Introduction

Discharge of pediatric patients following hematopoietic stem cell transplant (HSCT), also referred to as blood and marrow transplant (BMT), is a complex process and requires multi-disciplinary efforts to ensure patients/caregivers are prepared for discharge and transition to the outpatient setting. This period post-HSCT is tenuous as patients are medically complex, severely immunocompromised, and require a demanding medication regimen, with frequent dose modifications [1]. Miscommunication or decreased preparedness for discharge can lead to medication errors, lack of proper central-line care, and lack of adherence to social distancing and diet instructions, which can place patients at risk for life-threatening complications [2,3]. HSCT providers are reliant on patients/caregivers who are actively engaged in their care and recognize the importance of proper medication administration, nutrition, hydration, central line care, and the need to seek care for certain patient-reported symptoms [4]. Standardization of discharge for medically complex pediatric HSCT patients has the potential to decrease readmissions, improve caregiver readiness and confidence, and improve medication adherence.

Within the pediatric oncology division at a large hospital within the United States, there was no prior standardized process for HSCT discharges. The amount of education received from the period prior to the start of HSCT and, throughout the hospital admission, varied from day to day and by provider. Also missing was a process to evaluate whether parents/caregivers had a clear understanding of HSCT discharge information discussed.

Lack of a uniform approach to pediatric HSCT post-hospitalization care planning for patients and caregivers has the potential to cause patient harm. Due to this, a quality improvement (QI) project was performed to create a standardized discharge process to improve the overall transition from the hospital to the outpatient setting and increase understanding for patients and their families of post-transplant complications. This project was implemented for patients and caregivers within the pediatric oncology division of an academic children’s hospital over six months. This project aimed to establish a standard operating procedure regarding discharge processes and improve caregiver readiness for discharge.

### Review of the Literature

**Figure 1:**
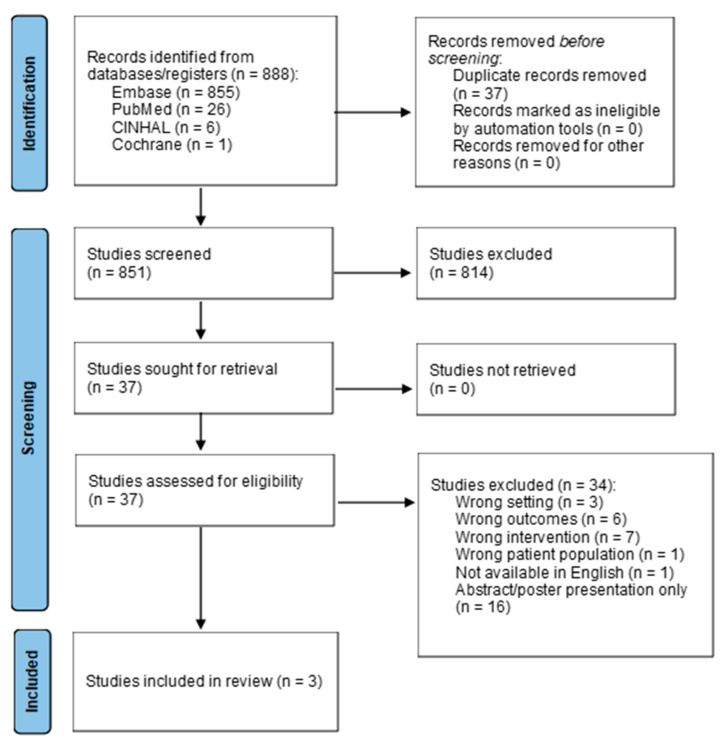
PRISMA Flowchart adapted from [6].

To determine the appropriate interventions, a literature search was performed in October 2024 to evaluate the approaches to discharge coordination and planning for pediatric patients undergoing HSCT using the databases of PubMed, CINAHL, Embase, and Cochrane. Search terms included the MeSH terms “bone marrow transplantation” or “stem cell transplantation” which was combined with “patient discharge” or “hospital to home transition” or “case management.” The results of these two search strategies were combined with a search for the MeSH terms “adolescent” or “child” or “infant”. Related keywords in titles and abstracts and truncations of search terms were included in the search strategies. Filters were applied to limit results to those published within the last 10 years (since 1 January 2014). Eight hundred and eighty-eight articles were discovered from the initial search after the removal of duplicates. Inclusion criteria included peer-reviewed articles published in the past 10 years including a population of pediatric HSCT patients aged 21 or younger, with an intervention regarding discharge care coordination, planning, or education. Articles were excluded if they involved adult or non-HSCT patients, were not related to the first discharge following HSCT admission or related interventions, were not available in English, or were solely an abstract or poster presentation. After preliminary screening, the 34 articles remaining underwent a full-text review, and only three articles were found to meet the inclusion criteria, as outlined in Figure 1. Appraisal of articles was performed via the Johns Hopkins Evidence-Based Practice Evidence Appraisal Tool [5].

This integrative review highlighted the scarcity of literature available on this topic, as summarized in Table 1. Three different approaches to pediatric HSCT discharges were described. Gladbach et al. [7] evaluated the feasibility of a “rooming in” time period where caregivers received comprehensive education, medication charts, and teaching handouts throughout the admission, and then for a 24 to 48 h period prior to discharge the caregiver stayed with the child and provided all care including medication administration, line care, and assistance with activities of daily living, under RN supervision with interventions as needed [7]. There were higher coping difficulty scores in those with higher numbers of home medications and this was also associated with lower readiness for discharge [7]. Although no significant differences were noted, overall scores for quality of discharge teaching related to the “rooming-in” group were high, indicating feasibility of this intervention [7]. West et al. [1] also utilized the concept of “rooming-in” as part of a discharge education bundle with three phases of implementation. Phase 1 consisted of education performed by nurses and pharmacists starting at pre-admission, Phase 2 was the requirements for a 24 h “room-in” with the designated caregiver providing all care in the hospital during that time, and Phase 3 consisted of the successful discharge from hospital to home/hotel [1]. With these interventions there was a decrease in 30-day readmissions for the post-implementation group [1]. Tang et al. took a different approach and evaluated the use of video-based BMT education which was found to be effective and noted higher proficiency scores [8]. They utilized a two-phase approach with Phase I consisting of providing a BMT handbook to parents with verbal teaching and Phase II adding a 14 min video including detailed discharge education [8].

## 2. Materials and Methods

Due to a lack of standardized approaches, this QI project built on the available literature in order to establish a new, consistent process for the transition of care for pediatric HSCT patients from hospital to home. This project utilized a pre-post intervention and longitudinal survey design. A historical practice group was compared to an implementation group, to look at self-reported ratings of caregiver readiness for discharge before and after interventions. A longitudinal approach was intended to be used to evaluate self-reported medication adherence ratings by caregivers. This program was developed and implemented within the Division of Pediatric Oncology of a children’s hospital in a large, urban academic center in the United States. Planning started in August 2023 and the first steps were implemented in March 2024 with data analyzed in September 2024. The multidisciplinary team at this center includes nurses (RNs), nurse practitioners (NPs), attending and fellowship-trainee physicians (MDs), overnight resident physicians, pharmacists (PharmDs), registered dieticians, social workers, child life specialists, BMT coordinator, nurse navigator, discharge coordinators, and support staff. Approximately 40 transplants are performed annually for patients aged 24 years and under, with malignant or non-malignant conditions. All patients and caregivers who received a HSCT at this center during the six-month implementation period received the interventions as a required part of their hospital stay.

**Figure 2:**
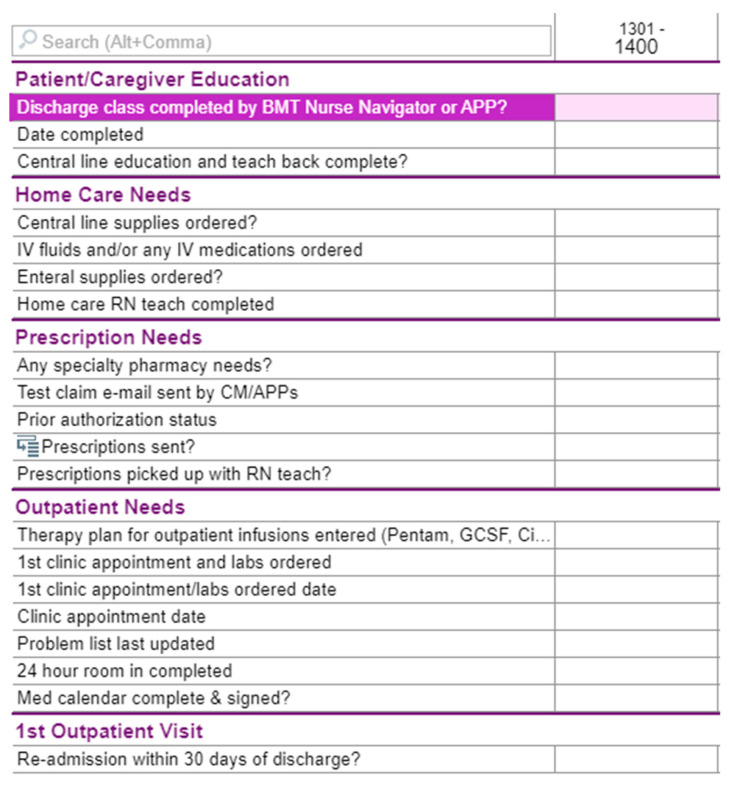
EMR discharge checklist.

The interventions for this QI project involved a multi-pronged approach with dedicated discharge QI team creation, education standardization, electronic medical record (EMR) integration, mandatory 24 h room-in, and individualized PharmD appointments. First, the multi-disciplinary discharge team was formed consisting of HSCT NPs, MDs, PharmDs, a nurse navigator, and nurse discharge coordinators. The involvement of the HSCT nurse navigator was pivotal in the implementation process as they serve as the standard point of contact for all HSCT patients before, during, and after transplant within this center. Next, center-available educational materials were standardized, to ensure focus on the HSCT process, immunosuppressive medications, diet and lifestyle requirements, and common post-transplant complications (e.g., graft versus host disease, fever in immunocompromised patients, etc.). Additionally, the discharge QI team developed an integrated checklist in the Epic^®^ EMR for all care team members to track the progress of the patient towards discharge (see Figure 2). This provided clarity for members of the multidisciplinary team, as well as allowing the patient and caregiver to accurately monitor their current status and progress towards discharge. A mandatory 24 h room-in for the designated caregiver prior to discharge was also implemented, where the caregiver owned all care and medication administrations towards the end of the hospitalization. The patient and caregiver continued to have a designated bedside RN for all questions and for legal purposes to oversee care and medication administration, as well as to scan all medications administered into the medication administration record (MAR) during the room-in. Due to psychosocial issues related to care of other children and/or work responsibilities by caregivers, some 24 h room-ins required breaking into two 12 h shifts to assure that caregivers could partake in this intervention. Finally, a standardized follow-up appointment was performed with a pharmD near discharge and at the first or second outpatient visit after discharge, where this provider assisted with filling medications into hospital-provided pill boxes and addressed any medication-related questions or adherence concerns. Following hospital discharge, surveys were administered to the primary caregiver.

In addition to demonstrating successful program creation and implementation, to assess the project’s impact, evaluation tools utilized surveys given via QR code embedded in Microsoft Forms™ at designated intervals. The Pediatric Transition Experience Measure (P-TEM) is a brief, 8-item, caregiver-reported outcome measure to assess hospital-to-home transition quality with documentation of validity for use in hospitalized children [9], and this was administered the week following discharge for the implementation group. This survey was also administered to patients who were discharged prior to the start of the project as a pre-implementation comparison, with timepoint of this varying between patients from weeks to a few months after discharge. All patients were to select how prepared they felt following discharge on a scale of 0, being the least prepared, to 100, being the most prepared. For ease of administration, surveys gave 0, 25, 50, 75, or 100 as potential answers. Patient reported medication adherence was to be evaluated utilizing a modified version of the Simplified Medication Adherence Questionnaire (SMAQ) which is a brief, six-item validated and reliable survey of self-reported medication adherence [10]. The sixth question, which asks about medication adherence over the past three months, was removed, and the modified SMAQ was intended to be administered to patients/caregivers post-implementation one week after discharge at approximately Day +30, one month after discharge at approximately Day +60, and two months after discharge at approximately Day +90 to aid in identification of barriers to medication compliance early and often. Due to difficulties in the administration of the SMAQ survey due to provider shortages, there were significant gaps in data, so this aim was dropped. Data from the PTEM was compared between groups and analyzed for statistical significance using the Mann–Whitney U Test via SPSS version 29. A *p*-value cut-off of <0.05 was considered statistically significant. **The Johns Hopkins IRB deemed this research a QI/Quality Assurance activity and thus the project adhered to all Johns Hopkins’ QI project policies. As such, consent was not required**. At the beginning of each survey, participants were informed that this information would be used to improve the division’s discharge process. They had the option to decline participation in the survey.

## 3. Results

From March to December 2024, caregivers for 12 patients were included in the post-implementation portion of this study, with nine patients pre-implementation. Of the 12 post-implementation patients, ten underwent allogeneic HSCT and two underwent autologous. Everyone in the post-implementation group was successfully able to complete all interventions, and caregivers completed a P-TEM survey. Caregiver responses to a P-TEM survey can be found in Table 2. There were no significant differences in caregiver-reported quality of care transitions following discharge after HSCT between the pre- and post-implementation groups. Most caregivers felt confident caring for their child at home in both groups and felt the written discharge instructions disseminated were helpful. All caregivers in the pre-implementation group and most in the post-implementation group reported feeling they had everything needed to provide care at home and that their follow-up provider was knowledgeable of their hospital stay on how to manage care after discharge. There was one caregiver in the post-implementation group who consistently reported lower scores, and later remarked to their care team that they would not have felt completely comfortable regardless of the amount of discharge preparation they received.

## 4. Discussion

This QI project sought to develop and implement a multi-disciplinary approach to better prepare patients and caregivers for the transition from hospital to home following HSCT, based on current literature recommendations. Based on the limited available literature, a discharge program was created and successfully implemented by a multidisciplinary team. Where there was previously no unified approach, the program created an organized approach with clear guidelines for roles and responsibilities among the multidisciplinary team ensuring complete and holistic education for all caregivers. Strong multi-disciplinary involvement was key to the success of this QI project. Key figures in the implementation and rollout of the project included NPs that provided continuity of care, the BMT nurse navigator that began education as early as the BMT evaluation appointment, and dedicated pharmDs that provided education on transplant medications and weekly check-ins for adherence assessments and pill box fills.

While the literature regarding approaches to discharge for pediatric HSCT patients is limited, there is available information on potential facilitators or barriers following discharge that when combined with this QI project’s findings could help develop best practices. Morrison et al. [11] conducted a grounded theory study on adolescents and young adults following HSCT to explore any facilitators or barriers to care after discharge. Patients and caregivers reported having printed instructions, a multidisciplinary source of information, and being able to track their milestones and progress toward discharge as being helpful [11]. While an EMR-integrated discharge checklist was utilized in this QI project, there is room to make this more patient/caregiver friendly. In West et al. [1]’s pediatric HSCT three-part discharge education bundle, top facilitators of the project included interdisciplinary support and engagement, standardization of unit-specific guidelines, and frequency of education. These interventions were vital to encouraging familial inclusion and increasing caregiver and patient demonstration of readiness. A caregiver demonstration of readiness was used during the 24 h room-in of this QI project, as were interdisciplinary support and standardization. With medically complex patients, caregiver involvement is paramount to successful post-discharge care, but this can lead to decreased quality of life and burnout for caregivers. Having care coordination that is tailored to meet specific patient and family needs can improve caregiver satisfaction with care, quality of life, caregiver independence, and overall quality of care coordination [12]. As noted in this QI project, the nurse navigator is paramount in ensuring that patients/family receive individualized education and support, and utilization of this role should be considered for other pediatric HSCT programs.

Regarding discharge practices within the greater pediatric hospitalized community, there is available literature discussing standardization to add to this project’s results. Wu et al. [13] published an article in 2016 discussing a successful multi-center QI project created by a multidisciplinary team of experts on discharge improvement. In this endeavor, a discharge toolkit was created and implemented in 11 hospitals, which included proactive discharge planning, standardized education processes, post-discharge treatment arrangement, and increased communication of discharge plan to the primary care provider [13]. The creation and use of a comprehensive discharge care coordination approach has been found to be successful at various centers with the care of medically complex pediatric patients [12,14,15,16]. Petitgout et al. [17] created a best practice alerts (BPA) tool integrated within the EMR to recognize children admitted who are medically complex and automatically assign a care coordinator to that patient. Having a previously established and strong continuity of care program comprising RNs and social workers was paramount to this BPA tool’s success. Other discharge coordination interventions that have been found to be successful for medically complex children include starting services during the inpatient admission [12,16] using a teach-back approach when providing education to parents and caregivers [15], planned integration and communication with caregivers within the EMR [16,18], and the utilization of readiness checklists [15]. These approaches could be tailored to meet the specialized needs of pediatric HSCT care and combined with this QI project’s newly established standardized discharge program to determine if these additional interventions add to patient/caregiver feelings of discharge preparedness.

Significant progress has been made but as with all QI, it is constantly evolving and there were various limitations to this study. The sample size for the project was small and therefore, generalizability is difficult to interpret. During implementation, there were less patients undergoing transplantation than in previous years. The project is a single center study and focuses on a niche patient population. Due to time constraints, there was not a pre- and post-confidence survey for parents that completed a 24 h room-in. The introduction of a mandatory room-in was a key intervention for this QI project by subjectively improving confidence, readiness, and decreasing medication error by allowing supervised practice; however, the impact was not objectively evaluated. Although there was excellent buy-in from stakeholders, it was difficult to ensure that the room-in could be completed in a consecutive 24 h due to familial needs outside of the hospital. Information on medication adherence via utilizing the SMAQ might contribute to future discharge projects and efforts to better incorporate this survey will be important. Pre-implementation and post-implementation surveys did not collect demographic data; therefore, comparisons to account for type of transplant, race/ethnicity, or zip code, variables that could affect the answers to survey questions. This center provides care to patients from all over the world speaking numerous languages; however, for this pilot we started with English-only standardized educational materials. Ongoing data/surveys are needed to establish significance. It was surprising that no differences between pre- and post-intervention groups were noted, but that lack of statistical significance does not take away from the need to standardize this process such that every patient/provider can benefit. This may speak to how well the multidisciplinary team already educated and prepared patients and caregivers for discharge prior to formalizing a process. This QI project was clinically significant as noted by the successful establishment of a standardized multidisciplinary discharge coordination process, and would benefit from a larger study, including qualitative analysis of feelings of preparedness.

## 5. Conclusions

An evidence-based, multi-disciplinary, multi-intervention approach throughout the hospital stay with a focus on discharge readiness can be successfully implemented for pediatric HSCT patients but the impact on caretaker confidence level in the transition from hospital to home is not yet clear. Consequently, by providing quality and individualized education as well as a trial run of home medical management via a room-in, caretakers may be able to leave the hospital prepared to care for the patient and powered with tools necessary to continue the desired care plan as laid out by the HSCT team. Critical to the success of any complex medical discharge program is stakeholder buy-in within a multi-disciplinary team. As with any QI project, many lessons have been learned and will be utilized for future implementation. Imagery will be created to visually depict progression to discharge throughout admission to patients and caregivers. This will be posted in each patient’s room and be updated throughout their HSCT hospitalization to serve as a reminder to patients and caregivers where they are in the transplant process and proximity to discharge. Additionally, with more resources, the project will expand to include standardized video-on-demand teaching for patients and caretakers to watch during the hospital stay and afterwards, as well as discharge education pamphlets in expanded languages to better assist non-English speaking families.

## Figures and Tables

**Table 1:** Table of evidence.

Author, Date, and Title	Study Type	Population, Size, and Setting	Intervention	Variables/Measures Used	Outcomes	Limitations	Evidence Level and Quality ^1^
Gladbach, C., Patton, L., and Xu, X. (2021). Transition From Hospital to Home Following HSCT: A Feasibility Study for “Rooming in” [7]	Feasibility, non-randomized, prospective vs. historical retrospective group	Pediatric BMT patients age 0–21 (*n* = 35 retrospective control, *n* = 35 with room-in intervention), BMT unit in a US pediatric academic medical center	Rooming-in intervention: Caregiver identified prior to BMT, caregiver receives comprehensive discharge education during admission (notebook pre-admit, med chart and teaching handouts/instructions during admission), then 24–48 h prior to discharge the caregiver provides all care (meds, line care, ADLs, etc.) with RN supervision	Discharge questionnaire packet completed on day of discharge. (Demographics form, Quality of Discharge Teaching Scale parent form, Readiness for Hospital Discharge Scale parent form). Post-discharge coping difficulty scale at discharge and day +30.	No significant difference in LOS and rooming in intervention. Negative association between longer LOS and readiness for discharge. Negative association between increased number of home meds and readiness. Less readiness associated with more phone calls. Positive association between quality of discharge ed and readiness. Good parent coping overall showed positive association with more meds. Higher coping difficulty scores associated with lower discharge readiness	Rooming-in is standard of care at center so control group is only retrospective patients. No ability to randomize. Only English-speaking families. Small sample size. Single study site.	Level III, Quality B
Tang, S., Landery, D., Covington, G., and Ward, J. (2019), The Use of a Video for Parents After Pediatric SCT [8]	Prospective, two phase pilot study	54 mothers of pediatric patients undergoing BMT (*n* = 17 with phase I teaching, *n* = 37 with phase II of teaching and intervention). BMT unit in an urban children’s hospital in the US	Phase I: BMT handbook provided by nurses to parents using verbal teaching and teachback method. Phase II: Video intervention added; 14 min video including information on home cleaning, when to notify medical team, GVHD, diet, visitor restrictions, and clinic visit expectations	Primary outcome measure: feasibility. Secondary outcome measure: Proficiency evaluation tool designed by content experts using 4-point Likert scale	Intervention was feasible as all completed phase II. Mothers in Phase II had higher proficiency scores on pre-discharge home cleaning, GVHD definition and symptoms, and diet restrictions. Phase I only participants had higher proficiency on when to call the BMT team after discharge. No differences on weekly home cleaning, visitor restrictions, and follow-up visits	Control group of standardized teaching alone vs. standardized teaching plus video intervention were evaluated sequentially with other practice changes occurring during this time. Small sample size. Single study site.	Level III, Quality B
West, Varnes, and Hudspeth (2023), Standardization of Pediatric HSCT Patient Discharge to Reduce Readmission Rates [1]	QI, Non randomized pre and post intervention	Pediatric BMT patients (intervention group *n* = 6, pre-intervention group *n* = 25). Hematology/Oncology unit of an academic children’s hospital in the US	Discharge Education Bundle with 3 specific phases—Phase 1: Prior to admission patient/caregiver provided with guidelines/unit expectations and multidisciplinary education including by staff RN and pharmacist utilizing teach-back method. Phase 2: 24 h rooming-in with caregivers providing all care. Phase 3: Completion of discharge from hospital to home/hotel	Primary outcome measure: readmission rate within 30 daysSecondary outcome measures: completion of 3 phases of discharge.	Decreased readmissions noted in intervention group compared to pre-intervention historical control. Process successfully implemented; New protocol was successfully created.	Small sample size. Non-randomized. Single study site. Large sample size discrepancy	Level V, Quality B

^1^ Grading per [5].

**Table 2:** Caregiver responses to P-TEM survey.

Question	Pre-Implementation Scale − *n* = x (%)	Post-Implementation Scale − *n* = x (%)	*p*-Value
I felt confident about how to care for my child at home	50 − 1 (11.1) 75 − 0 (0) 100 − 8 (88.9)	50 − 0 (0) 75 − 3 (25) 100 − 9 (75)	0.702
The written discharge instructions I was given were helpful for taking care of my child at home	50 − 0 (0) 75 − 1 (11.1) 100 − 8 (88.9)	50 − 0 (0) 75 − 1 (8.3) 100 − 11 (91.7)	0.917
I felt I had everything I needed to take care of my child at home after we left the hospital	50 − 0 (0) 75 − 0 (0) 100 − 9 (100)	50 − 0 (0) 75 − 2 (16.7) 100 − 10 (83.3)	0.554
I was able to easily contact my child’s hospital doctors with questions after we left the hospital	N/A ^1^	50 − 0 (0) 75 − 0 (0) 100 − 9 (100)	N/A ^1^
My preferences were considered when scheduling follow-up appointments for my child	N/A ^1^	50 − 0 (0) 75 − 1 (8.3) 100 − 11 (91.7)	N/A ^1^
The follow-up provider(s) knew what happened to my child in the hospital	50 − 0 (0) 75 − 0 (0) 100 − 9 (100)	50 − 0 (0) 75 − 1 (9.1) 100 − 8 (90.9)	0.766
The follow-up provider(s) knew how to manage my child’s medical care after we left the hospital	50 − 0 (0) 75 − 0 (0) 100 − 9 (100)	50 − 0 (0) 75 − 1 (8.3) 100 − 11 (91.7)	0.754

^1^ These questions were excluded from the pre-implementation group as the length of time following discharge was increased and at this point patients were being seen very frequently in clinics.

## Data Availability

The raw data supporting the conclusions of this article will be made available by the authors upon request.

## Public Involvement Statement

No public involvement in any aspect of this research.

## Guidelines and Standards Statement

This manuscript was drafted against the PRISMA 2020 guidelines [6] for systematic reviews.
